# Lipid Signaling During Gamete Maturation

**DOI:** 10.3389/fcell.2022.814876

**Published:** 2022-06-24

**Authors:** Sherif Mostafa, Nancy Nader, Khaled Machaca

**Affiliations:** ^1^ Medical Program, WCMQ, Education City, Qatar Foundation, Doha, Qatar; ^2^ Calcium Signaling Group, Research Department, Weill Cornell Medicine Qatar (WCMQ), Education City, Qatar Foundation, Doha, Qatar; ^3^ Department of Physiology and Biophysics, Weill Cornell Medicine, New York, NY, United States

**Keywords:** oocyte, egg, sperm, glycerolphospholipid, sphingolipid, ceramides, sterols

## Abstract

Cell lipids are differentially distributed in distinct organelles and within the leaflets of the bilayer. They can further form laterally defined sub-domains within membranes with important signaling functions. This molecular and spatial complexity offers optimal platforms for signaling with the associated challenge of dissecting these pathways especially that lipid metabolism tends to be highly interconnected. Lipid signaling has historically been implicated in gamete function, however the detailed signaling pathways involved remain obscure. In this review we focus on oocyte and sperm maturation in an effort to consolidate current knowledge of the role of lipid signaling and set the stage for future directions.

## 1 Introduction

**FIGURE 1:**
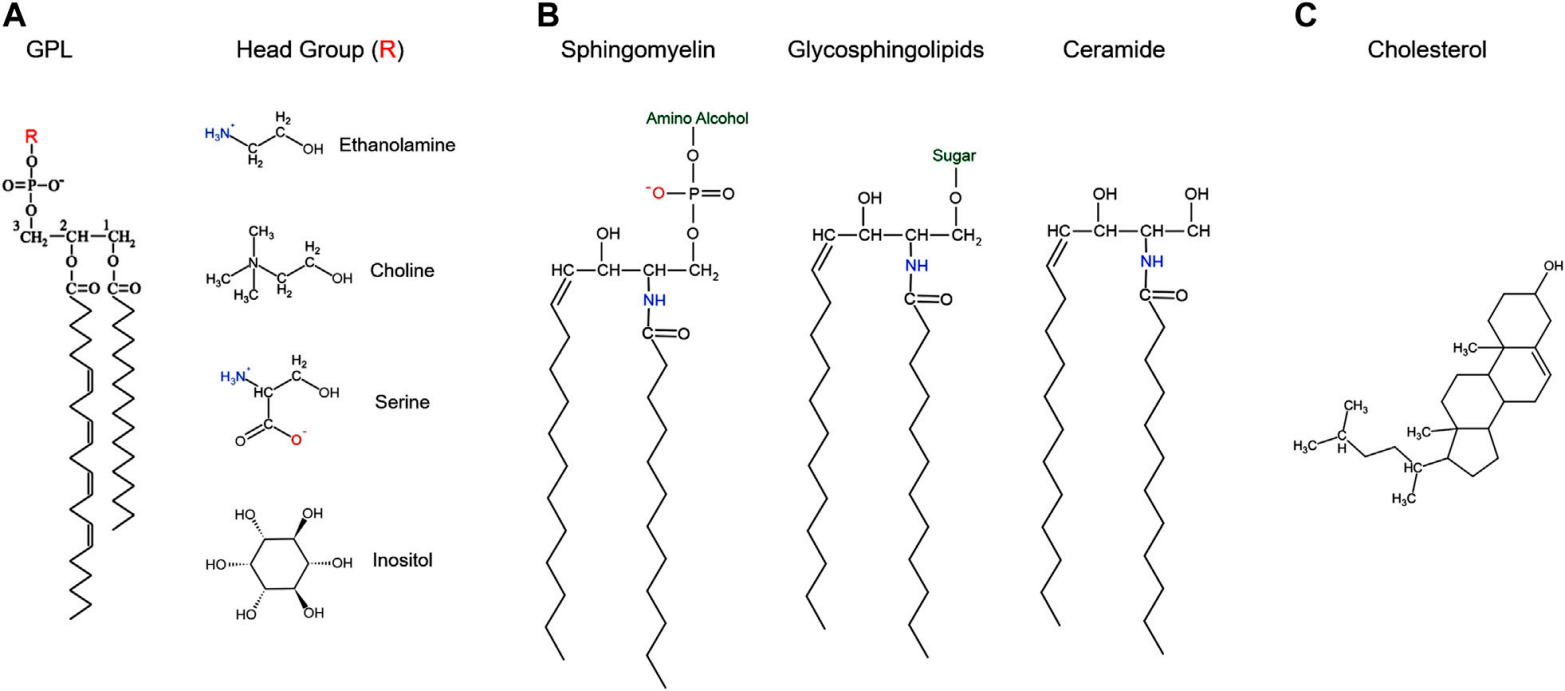
Structure of the major classes of cellular lipids.

In both gametes and somatic cells lipids play critical roles in cellular metabolism, structure, and signaling. The membranes of cellular organelles and the plasma membrane (PM) are highly dynamic lipid bilayers that allow for physical separation of biochemical processes within the cell that is essential for cellular homeostasis and function (Edidin, 2003; Pollard et al., 2016). Lipid bilayers are selectively permeable based on both their lipid and protein composition (Harayama and Riezman, 2018). Bilayers are composed of three broad lipid species: glycerophospholipids (GPL), sphingolipids, and sterols (Figure 1). These lipids are critical for membrane function, including its mosaic nature, fluidity, and vesicular trafficking. They solvate PM proteins, form microdomains with protein partners that act as signaling platforms, and are precursors for lipid second messenger that mediate cellular responses (Spector and Yorek, 1985; Lee, 2003; Arish et al., 2015; Pollard et al., 2016; Kim et al., 2019). Changes in the lipid composition lead to cellular switches that promote physiological and sometimes pathological changes (Casares et al., 2019; Grassi et al., 2020).

In addition to their signaling and structural roles, membrane lipids are important for cellular energy homeostasis. For example, intracellular lipid droplets are important for cellular metabolism as they store neutral lipids for energy, but also support storage of vitamins and signaling precursors in oocytes and somatic cells (Welte and Gould, 2017; Zhuan et al., 2020). The composition of lipid droplets has been shown to affect oocyte and embryo quality (Barrera et al., 2018). Lipids also act as precursors for steroids and eicosanoids in cumulus cells thus affecting the development of the cumulus oocyte complex (Nuttinck et al., 2008). This review focuses on the role of lipids as signaling intermediates as this area has been less well covered in the recent literature, especially in the case of the oocyte.

The ultimate function of gametes is to fuse at fertilization to produce a new zygote and ensure the continuity of the species. Gametes are highly differentiated cells that are not only haploid but specialized for the delivery of genetic material and for supporting embryonic development. Before becoming competent for fertilization both sperm and egg require a maturation period that endow them with the ability to fuse and initiate development. Clearly in the case of sperm but as well for eggs, lipid signaling plays a role in their maturation. Therefore, a better understanding of the role and regulation of lipid signaling in gamete maturation would provide important insights into both the physiology and pathophysiology of gamete function.

## 2 Cellular Lipids

Lipids can be classified into three broad categories: glycerophospholipids (GPL), sphingolipids, and sterols (Arish et al., 2015; Pollard et al., 2016) (Figure 1).

### 2.1 Glycerophospholipids

GPLs can be divided into four classes based on their headgroups: phosphatidylethanolamine (PE), phosphatidylcholine (PC), phosphatidylserine (PS), and phosphatidylinositol (PI) (De Craene et al., 2017). These lipids are made up of three parts: a three-carbon glycerol backbone, a phosphoric acid group attached to the C3 hydroxyl group with an ester bond linking to different head groups, and two long-chain fatty acids attached to the C1 and C2 carbons with ester bonds (Figure 1A). The fatty acid chains can be saturated or unsaturated and therefore contribute to PM fluidity as unsaturated fatty acids tend to reduce fatty acid chain packing in the membrane and increase its fluidity (Kanno et al., 2007). The head group dictates the overall charge of the molecule: positive for PE, negative for PS and PI, or neutral for PC (Figure 1A) (Pollard et al., 2016). These charged groups are important in the function and geometric shape of each GPL and play a role in dictating their distribution on the PM (Daleke, 2007). For example, given the positive charge of PE, when hydrated at physiologic pH, PE can organize itself into hexagonal phase-prone lipid microdomains that are important in recruiting amphitropic signaling proteins like G-proteins (Vögler et al., 2004; Torres et al., 2020). GPLs are amphipathic molecules that make up the PM bilayer, with asymmetric inner and outer leaflets (Farooqui et al., 2000; Fadeel and Xue, 2009; Arish et al., 2015). Typically, PS, PE and PI are enriched in the inner leaflet, whereas PC and sphingolipids are more abundant in the outer leaflet (Van Meer et al., 2008). This differentiation in inner and outer leaflet components contributes to many processes including formation of lipid rafts and secondary messenger lipids that play important roles in cell signaling (De Kroon et al., 2013; Sunshine and Iruela-Arispe, 2017).

### 2.2 Sphingolipids

**FIGURE 2:**
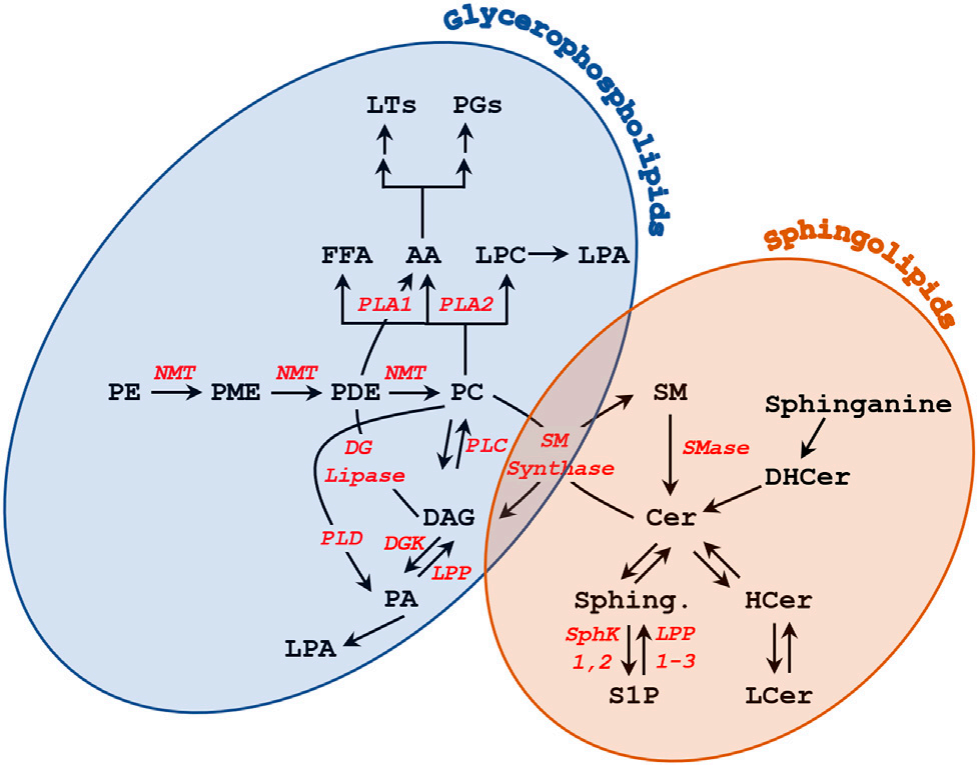
Metabolism of glycerophospholipids and sphingolipids.

Sphingolipids are composed of a sphingosine backbone–derived from serine and a fatty acid–attached by an amide bond to a fatty acid chain (Pollard et al., 2016) (Figure 1C). Sphingolipids are classified into three classes based on the head group added on the C1 sphingosine: ceramides, glycosphingolipids, and sphingomyelin (Figure 1B). Ceramides contain a hydroxyl (OH) group on C1 and is an important constituent molecule of other sphingolipids like glycosphingolipids, sphingomyelin, ceramide-1-phosphate, and even sphingosine (Castillo et al., 2016) (Figure 1B). Ceramide is converted into sphingosine by ceramidases, and a phosphate group can be added by sphingosine kinase to produce sphingosine-1-phosphate (S1P) (Castillo et al., 2016). S1P has been shown to bind to G-protein coupled receptors (GPCR) on the PM and activate different signaling pathways, that include but are not limited to stimulating cell survival and proliferation while inhibiting cell death (Monick et al., 2004; Arish et al., 2015; Castillo et al., 2016). Glycosphingolipids are produced by adding different sugar groups at the C1 position. Cerebrosides contain a single sugar moiety like glucose or galactose; lactosylceramide contain a lactose group; gangliosides contain oligosaccharides and negatively charged sialic acid groups; whereas sulfatides contain a sugar and a sulfate group (Castillo et al., 2016; Kim et al., 2016; Kim et al., 2019). Gangliosides play a role in cell differentiation and different signaling pathways like epidermal growth factor receptor and fibroblast growth factor receptor signaling (Kim et al., 2008; Kim et al., 2016). These gangliosides also play roles in oocyte and sperm motility, which will be discussed in further details. Sphingomyelin is produced by adding a phosphate group esterified with an amino alcohol (e.g. choline or ethanolamine) (Figure 1B). These sphingomyelins can be converted back to ceramides by sphingomyelinases (SMase) (Figure 2) and support microdomain formation and/or alter fluidity and morphology of the PM (Jafurulla et al., 2008; Arish et al., 2015).

### 2.3 Sterols

The third major class of lipids are the sterols, of which cholesterol is the major representative in mammalian PMs (Figure 1C) (Barenholz, 2002; Pollard et al., 2016). Cholesterol is made up of a rigid nonpolar four ring steroid structure that allows it to wedge itself deep into the lipid bilayer, a hydrocarbon side chain and a hydrophilic hydroxyl group that is oriented towards opposite surfaces (Dufourc, 2008; Pollard et al., 2016) (Figure 1C). Cholesterol is important in many cellular processes including PM fluidity, signal transduction, and modulating membrane lipid and protein functions (Liu and Ding, 2017). In fact, the PM of eukaryotic cells contains between 40 and 90% of the total cellular cholesterol. Cellular cholesterol levels are tightly maintained between inaccessible cholesterol in PM lipid-rafts and free cholesterol in the intracellular pool. The flux of cholesterol from the PM to the endoplasmic reticulum (ER) can happen via endocytic vesicles, or via non-vesicular traffic by lipid transfer proteins at membrane contacts sites (Lange et al., 2014; Litvinov et al., 2018). Cholesterol further affects membrane thickness (Nezil and Bloom, 1992), which would be important for enriching for specific proteins in membrane subdomains where cholesterol is concentrated through hydrophobic matching. It is energetically unfavorable for the hydrophobic transmembrane domains of integral membrane proteins to be exposed to the aqueous environment or for hydrophilic portions of the protein to be embedded within the hydrophobic core of the bilayer. This has given rise to the concept of hydrophobic matching where the length of the hydrophobic transmembrane domains of integral membrane proteins need to match membrane thickness, which can be affected by cholesterol (Killian, 1998). Finally, cholesterol reduces the voids within the bilayer thus making it more compact and as such reducing its membrane permeability to water and other lipophilic and neutral small molecules (Shinoda, 2016).

## 3 Roles of Lipid Signaling in Oocytes

Vertebrate oocyte maturation prepares fully grown immature oocytes in the ovary for fertilization and is as such a requirement for the initiation of development (Machaca, 2007; Nader et al., 2013). Immature oocytes are arrested in prophase I of meiosis for extended periods of time that can be up to several decades in humans for example. This meiotic arrest is released through hormonal triggers resulting in the initiation of maturation and the transition of the oocyte to metaphase II of meiosis, at which stage it arrests again awaiting fertilization (Morrill and Kostellow, 1998; Spricigo et al., 2015; Matsubara et al., 2019). A Ca^2+^ signal at fertilization results in the completion of meiosis with the extrusion of the second polar body and the transition to mitotic cell division to initiate embryonic development (Machaca, 2007). Oocyte maturation encompasses not only the reductionist meiotic divisions that lead to the generation of a cell with a haploid DNA complement, but also importantly dramatic remodeling of signaling components and organelle distribution to produce a fertilization competent egg able to support embryonic development (Nader et al., 2013; Fan and Sun, 2019; Matsubara et al., 2019).

Changes in oocyte lipid composition can have significant impact on crucial biological functions. Therefore, studying the role of lipid signaling in oocytes and their associated cells -like cumulus cells- can expand our knowledge of how to define the oocyte’s developmental competency and quality (Auclair et al., 2013; Vireque et al., 2017).

### 3.1 Glycerophospholipids During Oocyte Maturation

A role for lipid signaling in oocyte maturation has been suggested early on in the literature from studies in amphibian oocytes. It was shown that progesterone (P4), the maturation inducing hormone in the frog, stimulates methyl incorporation into glycerophospholipids (GPLs) in both *Xenopus* (Godeau et al., 1985) and *Rana* oocytes (Chien et al., 1986). This occurred within seconds to minutes of P4 exposure and resulted in incorporation into phosphatidylcholine (PC), since sequential methylation of phosphatidylethanolamine (PE) and phosphatidylmonomethylethanolamine (PME) leads to formation of PC (Godeau et al., 1985; Chien et al., 1986). The increased N-methylation was also associated with the activation of sphingomyelin (SM) synthase as radiolabeled methyl groups were incorporated into SM through PC (Morrill et al., 1994). Most of the increased methylation was associated with the PM (Chien et al., 1991). In parallel, inhibitors of N-methylation blocked entry into meiosis in *Rana* oocytes (Chien et al., 1991). However, the signaling pathways through which lipid methylation supports oocyte maturation are not known.

**FIGURE 3:**
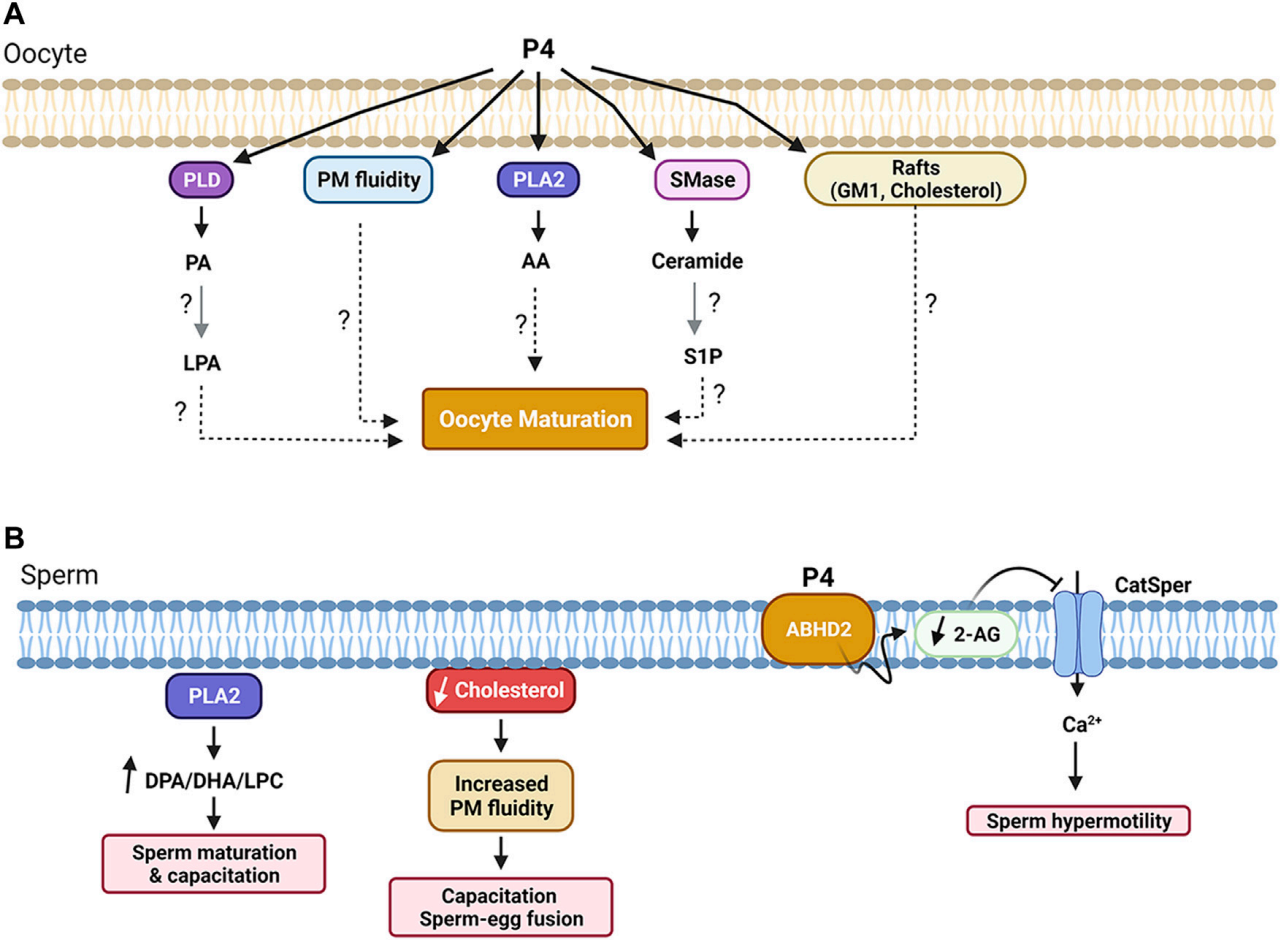
Summary of lipid-dependent signaling during oocyte **(A)** and sperm **(B)** maturation. See text for details. Figure created with BioRender.com.

P4 exposure also led to an increase in PM order and a decrease in PM fluidity in *Rana pipiens* oocytes (Morrill et al., 1989; Morrill et al., 1993), which was implicated in the ability of the oocyte to resume meiosis (Figure 3A) (Morrill et al., 1993). Furthermore, P4 led to rapid changes in PE, PI, and PC content on the PM, along with other PM lipids like sphingomyelin, together with releasing lipid second messengers, indicative of activation of phospholipases and sphingomyelinases (Kostellow et al., 1996; Morrill and Kostellow, 1998) (Figure 3A).

Collectively these studies argue that oocyte maturation is associated with rapid remodeling of the oocyte GPL content and suggest a functional role for this remodeling as it affected the ability of the oocyte to mature when blocked. However, how these changes in lipid composition mediate downstream signaling to release the oocyte meiotic arrest and initiate maturation remains elusive. Oocyte maturation in the frog is a slow process that takes hours to complete. It involves translational control through the regulation of polyadenylation and the activation of multiple signaling cascades that culminate in the activation of maturation promoting factor (MPF, Cdk1), the main kinase that drives entry into M-phase (Machaca, 2007). It is therefore significant that changes in plasma membrane lipids are observed on the time frame of minutes following P4 exposure, arguing that they may be involved in the early signaling to release the oocyte meiotic arrest and induce oocyte maturation.

#### 3.1.1 Phospholipases and Lysophosphatidic Acid

*Xenopus* oocyte maturation can be induced either by the physiological stimulus P4 or by insulin (Stith and Maller, 1987). This raised interest in investigating the role of different phospholipases in the process, in particular PLCs as Ca^2+^ transients have also been implicated in inducing oocyte maturation in early studies. PLCs hydrolyze phosphatidylinositol 4,5-bisphosphate (PIP2) into inositol 1,4,5-trisphosphate (IP_3_) and 1,2 diacylglycerol (DAG). IP_3_ binds to IP_3_ receptors on the ER membrane inducing a cytosolic Ca^2+^ rise. Early reports argued that a cytosolic Ca^2+^ rise was sufficient to induce oocyte maturation (Wasserman and Masui, 1975; Moreau et al., 1976; Schorderet-Slatkine et al., 1976). In support of these findings, injecting the oocyte with high concentrations of Ca^2+^ buffers inhibited oocyte maturation (Moreau et al., 1976; Duesbery and Masui, 1996). This was later found to be due to the ability of Ca^2+^ buffers such as BAPTA to chelate transition metals and not due to their Ca^2+^ sequestering function (Sun et al., 2007). Furthermore, inducing Ca^2+^ release by directly injecting IP_3_ was insufficient by itself to release the oocyte meiotic arrest (Picard et al., 1985; Stith and Maller, 1987). Also changes in PI metabolites in amphibian oocytes in response to P4 have been monitored with conflicting reports (Varnold and Smith, 1990; Chien et al., 1991; Stith et al., 1992; Morrill and Kostellow, 1999). Consistently and in contrast to earlier reports, depriving the oocyte completely of either Ca^2+^ release from intracellular stores or influx from the extracellular space did not prevent entry into meiosis in response to progesterone, but rather accelerated oocyte maturation kinetics (Sun and Machaca, 2004). This shows that not only are Ca^2+^ signals not required for oocyte maturation but that they negatively regulate the process. Ca^2+^ signals were however needed for completion of meiosis I as oocytes deprived of Ca^2+^ signals could not complete meiosis I (Sun and Machaca, 2004). Similar results were obtained in mouse oocytes (Carroll and Swann, 1992; Tombes et al., 1992). Collectively these findings argue against a role for Ca^2+^ signals or phosphatidylinositol metabolism in initiating oocyte maturation.

However, in addition to generating a Ca^2+^ transient, PLC also produces DAG that activates PKC. Hence PLC could initiate maturation through DAG-PKC activation. Indeed, treating oocytes with the DAG analog 12-O-tetradecanoylphorbol 13-acetate (TPA) induced maturation in the absence of P4 (Stith and Maller, 1987). But this effect seems to be PKC independent, as PKC injection did not release oocyte meiotic arrest (Stith and Maller, 1987). Maturation inducing hormones were also associated with an increase in sn-1,2-DAG levels at 15 min after addition, although immediately after addition (5 s) P4 led to a decrease in DAG levels (Stith et al., 1991; Stith, 2015). P4 also resulted in increased levels of sn-1,2-DAG in *Rana* (Chien et al., 1991) and *Xenopus* oocytes (Wasserman et al., 1990). In contrast, Varnold and Smith observed a decrease in DAG of >50% 5 min after the addition of P4 (Varnold and Smith, 1990), but this was later shown to be a technical artifact (Wasserman, 1992). Later metabolic and pharmacological inhibitors studies in *Rana* argued against PLC activation in response to P4 but rather favored PLD activation resulting in phosphatidic acid (PA) production, which importantly preceded the rise in DAG arguing for a P4-dependent PLD activation (Kostellow et al., 1996) (see Figure 2 and Figure 3A). PLD activation was also supported in *Xenopus* oocytes in response to insulin, which increase phosphatidic acid (PA) levels within 5 min (Holland et al., 2003). The PLD pathway can be a source of DAG as well during oocyte maturation as PA can be converted in DAG through the action of either lipin phosphatidate phosphatases or lipid phosphate phosphatases (LPPs). LPPs in addition to PA can act on other lipid phosphate substrates (Zhang and Reue, 2017). Several lines of evidence support a role for lipin during fertilization in *Xenopus*, which is also associated with an increase in DAG levels (Stith, 2015). Furthermore, PA can activate both Src kinase and PLCγ during *Xenopus* fertilization (Bates et al., 2014). There is an extensive body of evidence supporting a role for Src-related tyrosine kinases in activating PLC*γ* during *Xenopus* fertilization through lipid raft-dependent signaling leading to the explosive Ca^2+^ release that triggers egg activation (Sato et al., 1999; Sato et al., 2000; Sato et al., 2002; Mahbub Hasan et al., 2005; Sato et al., 2006). However, a detailed discussion of the role of Src-PLC*γ* during *Xenopus* fertilization is beyond the scope of this review, and has been expertly reviewed elsewhere (Sato et al., 2006; Stith, 2015). Therefore, the preponderance of the evidence argue for an increase in DAG levels in response to progesterone in amphibian oocytes due to PLD activation (Figure 3A).

There is also evidence supporting activation of phospholipase A2 (PLA2), as on the longer time frame of 1–2 h post P4, arachidonic acid (AA) was produced primarily from PC and to a lesser extent PE (Chien et al., 1986) (Figures 2, 3A). Consistent with the potential role of lipases in the initiation of oocyte maturation, injection of PLA2, PLC, or PLD was sufficient to release oocyte meiotic arrest in *Xenopus* in the absence of P4 (Carnero et al., 1994). Although this does not address which of these lipases is involved in the physiological release of oocyte meiotic arrest, it argues for a role for lipid signaling, in the induction of oocyte maturation. Collectively these studies show that exposure of amphibian oocytes to the maturation hormone P4, is associated with remodeling of cellular lipids with biochemical (generation of potential second messengers) and structural (decreased PM fluidity) changes.

The activation of phospholipases was coupled to the production of lipid second messengers including lysophosphatidic acid (LPA), AA, DAG, and PA (Carnero et al., 1994). Also, insulin exposure led to an increase in LPA as well as PA in the oocyte (Holland et al., 2003). *Xenopus* oocytes do express several LPA receptors, so it is possible that LPA produced during maturation acts in an autocrine fashion to stimulate or support oocyte maturation (Guo et al., 1996; Liliom et al., 1996). LPA is of particular interest in this case as is has been implicated in oocyte maturation in other species. This is not surprising given the pervasive role of LPA in cellular signaling and cell proliferation (Jarvis et al., 1984; Hinokio et al., 2002; Boruszewska et al., 2015). LPA has been shown to enhance oocyte maturation rates in bovine oocytes during *in vitro* maturation, in addition to increasing the mRNA abundance of specific factors, such as OCT4, SOX2, IGF2R in the oocyte and subsequent blastocyst (Boruszewska et al., 2015). The levels of these transcripts in oocytes are directly related to their developmental competence (Boiani et al., 2002; Keramari et al., 2010; Boruszewska et al., 2015). Furthermore, LPA has been shown to increase anti-apoptotic factors such as BAX and BCL2 and thus decrease the rates of apoptosis in the cumulus-oocyte-complex (COCs), which are necessary for the growth, viability, and maturation of the oocyte (Yang and Rajamahendran, 2002; Boruszewska et al., 2015). Also, LPA increased GLUT1 and PFKP expression in cumulus cells and hence enhanced glucose uptake in bovine COCs, which was the first evidence demonstrating that LPA directs cumulus cells to employ glycolysis to breakdown glucose into pyruvate or lactate, which are energy substrates used by oocytes during maturation (Cetica et al., 1999; Cetica et al., 2002; Boruszewska et al., 2015). LPA has also been shown to protect the COCs and the granulosa cells from zearalenone toxicity, a mycotoxin produced from *Fusarium* Fungi that can cause deleterious effects on human and animal reproductive organs, as well as inhibiting oocyte maturation (Massart et al., 2008; Bandera et al., 2011; Lai et al., 2018).

Moreover, LPA stimulated both nuclear and cytoplasmic maturation of immature golden hamster oocytes by activating cumulus cells (Hinokio et al., 2002), as well as promoting meiotic maturation in mouse oocytes (Komatsu et al., 2006). In particular, LPA participates in oocyte meiotic progression by increasing the levels of maturation-promoting factor (MPF) and mitogen-activated protein kinase (MAPK) activity in porcine oocytes (Zhang et al., 2015). Treating porcine oocytes with LPA has also been shown to reduce the frequency of polyspermy during fertilization (Zhang et al., 2015). Therefore, LPA represents a candidate GPL metabolite involved in oocyte maturation in several species.

It is however important to note for this discussion that detection of changes in the levels of lipid metabolites during oocyte maturation cannot be taken as proof of a functional role especially given the highly interconnected nature of lipid metabolism, stability of particular intermediates to allow their accurate detection, and potential localized action of metabolites which can be significant functionally but hardly detectable at the whole cell level. So, the findings of changes in the levels of lipid metabolites (up or down) in the absence of functional data supporting the role of a specific pathway must be considered with caution.

Also, one needs to be careful in interpreting the effects of hyper-activation of particular pathways on oocyte maturation, such as following injection of phospholipases or high concentration of lipids. The induction of oocyte maturation is ultimately mediated by tipping the stable balance between multiple signaling networks that maintain oocyte meiotic arrest to activate MPF (Cdk1), which is the main driver of the G2/M transition. Modulators may induce maturation by activating or inhibiting the kinases/phosphatases that maintain MPF inactive without necessarily operating through the physiological pathways that induce maturation. Furthermore, lipid injection can alter the structure and permeability of cellular membranes leading to unwanted leakage of Ca^2+^ or other metabolites that would indirectly affect oocyte maturation thus confounding the results obtained. Therefore, an experimentally more convincing approach to implicate a particular pathway in oocyte maturation is to show that its inhibition prevents maturation in response to the physiological hormone P4.

Finally, the field of lipid signaling has seen significant technical advances that allow for more accurate evaluation of the profiles of different lipid classes and species within a class. Mass-based assays for lipids such as mass spectrometry and high-performance liquid chromatography (HPLC) coupled to evaporative light scattering detection (ELSD) are more trustworthy with less variability than the older approaches such as thin layer chromatography (TLC) and turnover of metabolic labels. TLC may be associated with artifacts due to variability in sample preparation and storage from one experiment to the next due to lipid derivatization and oxidation during the separation process. Also, the use of home-made TLC plates in the earlier studies limits reproducibility. However, the improved methodology of high-performance thin-layer chromatography (HPTLC) still provides a powerful analytical tool to study lipid metabolism (Fuchs et al., 2011). ELSD is also sensitive to the number and type of polar functional groups in different lipids as well as to fatty acid chain length and degree of unsaturation (Melis et al., 2021). Furthermore, stable metabolic labeling studies to assess lipid signaling may also be fraught with technical difficulties especially in the absence of controls showing equilibrium labeling of the particular precursor pool of interest. Therefore, one needs to consider these technical limitations while assessing lipid signaling analyses. So, measurements of fluctuations in lipid levels need to be coupled to functional studies confirming the suspected role for a particular pathway.

Most of the studies tackling GPL signaling have focused on changes of particular metabolites without further defining downstream signaling by lipid mediators. The availability of mass spectrometry approaches that allow lipidomics analyses at the whole cell level warrants a reassessment of the early changes in lipid signaling during oocyte maturation to define their role and requirement for oocyte maturation.

### 3.2 Sphingolipids in Oocyte Maturation

#### 3.2.1 Ceramide

Sphingolipid metabolism has also been implicated in the activation of oocyte maturation in response to P4 in amphibian oocytes. In *Xenopus*, P4 exposure was associated with a time- and concentration-dependent increase in ceramide (Cer) and a decrease in sphingomyelin (SM) indicating the activation of sphingomyelinases (SMase) (Figure 2 and Figure 3A) (Strum et al., 1995). Consistently, exposing oocytes to SMases resulted in P4-independent maturation that required protein translation (Varnold and Smith, 1990; Strum et al., 1995; Coll et al., 2007). Maturation was also induced by injection of different sphingolipids, including sphingosine and Cer (Strum et al., 1995; Morrill and Kostellow, 1998). Furthermore, Cer exposure of *Rhinella* oocytes led to oocyte maturation (Buschiazzo et al., 2011). However, these findings could not be replicated by others, where exposure to Cer did not induce maturation in *Xenopus* oocytes (De Smedt et al., 1995). Similar findings were reported in the seasonal breeder *Rana pipiens* where exposure to Cer or injection of SMase was not able to induce oocyte maturation; but this was the case only during the breeding season, arguing for a requirement for prior exposure to a priming signal before Cer is capable of inducing maturation by itself (Morrill and Kostellow, 1998). In agreement with this conclusion, frogs used in the *Xenopus* studies to investigate the role of SMase and Cer were primed by gonadotropin injection before oocyte isolation (Strum et al., 1995). These data argue that oocyte maturation is associated with changes in Cer levels, but that these changes are not the primary trigger nor are they sufficient by themselves for the induction of maturation. One potential explanation is that changes in the levels of Cer are a consequence of alterations in other lipid metabolic pathways especially that the metabolism of GPLs is linked to that of sphingolipids through the activity of SM synthase (Figure 2).

Like other lipid metabolites, ceramides have been proposed to affect oocyte maturation through modulating Ca^2+^ signaling. Kobrinsky *et al.* argued for a functional crosstalk between ceramide metabolism and phosphoinositide signaling. They observed an increase in intracellular Ca^2+^ release when *Xenopus laevis* oocytes underwent ceramide-induced maturation (Kobrinsky et al., 1999). The increase in ceramide levels was associated with higher levels of IP_3_ via Gαq/11 and PLCβ activation (Kobrinsky et al., 1995; Kobrinsky et al., 1999). Furthermore, downregulating the expression of the IP_3_ receptor in *Xenopus laevis* oocytes delayed progesterone-induced oocyte maturation (Kobrinsky et al., 1995). However, as discussed above Ca^2+^ release has been shown to be dispensable for the release of oocyte meiotic arrest (Sun and Machaca, 2004), so it is unlikely that these lipid changes exert their action on oocyte maturation through Ca^2+^ signals. The effect of the downregulation of the IP_3_ receptor on maturation may be due to overall oocyte function as Ca^2+^ signaling is required for many homeostatic functions. So, downregulation of the IP_3_ receptor could affect the general health of the oocyte and thus slow down oocyte maturation, independent of a direct role for Ca^2+^ signals in releasing oocyte meiotic arrest.

Progesterone initiates oocyte maturation in *Xenopus* oocytes by binding to the membrane progesterone receptors (mPRs), and not the classical nuclear progesterone receptor, which induces a non-genomic signaling cascade to initiate maturation (Josefsberg Ben-Yehoshua et al., 2007; Nader et al., 2020). mPRs belong to the PAQRs (progestin and adipoQ receptors) family (Moussatche and Lyons, 2012), and share sequence similarity with alkaline ceramidase (AlkCer), which is a 7-transmembrane receptor that catalyzes reversible ceramide de-acylation to sphingosine (Kim et al., 2006; Villa et al., 2009; Moussatche and Lyons, 2012). This similarity might explain the alteration in PM lipid contents upon progesterone treatment (Morrill et al., 1989; Varnold and Smith, 1990; Strum et al., 1995), although there is no definitive proof that mPR*β* in the frog oocyte functions as a ceramidase. Recently, progesterone was found to induce clathrin-dependent endocytosis of mPRβ into signaling endosomes, which is required for oocyte maturation in *Xenopus* oocytes (Nader et al., 2020). Moreover, the action of progesterone on the plasma membrane has been proposed to occur on specialized membrane rafts like caveolae (Buschiazzo et al., 2011) that are rich in different classes of lipids, including cholesterol and GM1, and different classes of proteins, such as Src family kinases, Ras, adenylyl cyclase, and PIK3 (Song et al., 1996; Ostrom et al., 2001; Sadler, 2001; Chen et al., 2007). Ceramide can selectively displace cholesterol, Src, caveolin-1, and GM1 from membrane rafts to other non-raft areas, allowing cholesterol to be rapidly internalized, which affects the molecular and biophysical properties of the raft and the PM (Buschiazzo et al., 2011). There is however no experimental evidence for such a mechanism operating in the oocyte.

#### 3.2.2 Sphingosine and its Derivatives

In a similar fashion to ceramide, microinjection of sphingosine released meiotic arrest and induced oocyte maturation (Varnold and Smith, 1990; Strum et al., 1995). As sphingosine is a downstream metabolite of ceramide this argues that ceramide may not be the biologically active substance that is inducing maturation but rather that it is its downstream metabolites. In that context, sphingosine can be phosphorylated by sphingosine kinase to produce sphingosine-1-phosphate (S1P), which acts extracellularly, as it is extruded from the cell via an S1P transporter. S1P binds to and activates S1P receptors, which are GPCRs (Okamoto et al., 1998; Zondag et al., 1998; Villa et al., 2009). Given that various S1P receptors link to multiple second messengers pathways, S1P can branch out into activating various pathways from phospholipases to cAMP signaling, which may impact oocyte maturation (Durieux et al., 1993; Noh et al., 1998; Uhlenbrock et al., 2002). In *Xenopus* oocytes, S1P is mostly linked to Ca^2+^ signaling (Durieux et al., 1993; Noh et al., 1998). S1P has been implicated in oocyte maturation in mice, where oocytes collected in the presence of S1P showed a consistent and significant delay in oocyte maturation (Hinckley et al., 2005). However, adding S1P to the oocyte maturation medium was found to be beneficial to the development of immature mouse oocytes (Jee et al., 2011). S1P has also been shown to prevent apoptosis of mice oocytes exposed to chemotherapeutic damage (Morita and Tilly, 2000), and it protected bovine oocytes from heat shock during maturation (Roth and Hansen, 2004).

#### 3.2.3 Gangliosides

Gangliosides are complex glycosphingolipids that have been implicated in oocyte maturation through modulation of different processes, including epidermal growth factor receptors (EGFR), lipid rafts, and Ca^2+^ signaling (Kim et al., 2019). EGFR is a receptor tyrosine kinase (RTK) that is involved in many cellular processes including cell proliferation and prevention of apoptosis. Ligand binding results in EGFR dimerization and tyrosine autophosphorylation forming docking sites for SH2 domain containing signaling molecules, causing signal propagation. EGF signaling has been implicated in the release of oocyte meiotic arrest in mammalian follicles through paracrine signaling (Jaffe and Egbert, 2017). Cumulus cells surrounding the oocyte produce cGMP, which diffuses through gap junctions into the oocyte to maintain the long-term meiotic arrest. Activation of EGFR leads to lower cGMP levels in cumulus cells -and by extension the oocyte- thus supporting oocyte maturation (Jaffe and Egbert, 2017).

The GM3 ganglioside is able to inhibit EGFR autophosphorylation, without affecting ligand binding, thus preventing EGR signaling (Miljan et al., 2002). In porcine cumulus-oocyte complexes (COCs), exposure to exogenous GM3 inhibited oocyte maturation, cumulus cell expansion, and initiated apoptosis (Park et al., 2017). In addition, preimplantation mice embryos undergoing apoptosis have high levels of GM3 (Ju et al., 2005), and GM3 levels decrease during oocyte maturation and early embryonic development to prevent apoptosis (Kwak et al., 2003; Kim et al., 2019).

Another ganglioside implicated in oocyte maturation is GM1, which along with sphingomyelin and cholesterol, is enriched in PM membrane lipid rafts in *Rhinella arenarum*, *Xenopus laevis,* and mouse oocytes (Sato et al., 2002; Buschiazzo et al., 2011; Sato et al., 2011; Kim et al., 2019). The GM1-dependent organization of lipid raft microdomains on the oocyte PM impacts sperm attachment and fertilization in humans (Van Blerkom and Caltrider, 2013). Moreover, GM1-rich microdomains contain the plasma membrane Ca^2+^-ATPase (PMCA), a Ca^2+^ pump that extrude Ca^2+^ out of the cell and is internalized into an intracellular vesicular pool during *Xenopus* oocyte maturation (El-Jouni et al., 2005; El-Jouni et al., 2008). PMCA is internalized by raft-dependent endocytosis, which is slowed down when cholesterol is depleted from the PM (Kirkham and Parton, 2005; El-Jouni et al., 2008). PMCA internalization during oocyte maturation does not involve clathrin-dependent endocytosis and importantly contributes to the remodeling of Ca^2+^ signaling pathways during oocyte maturation (El-Jouni et al., 2008). A Ca^2+^ signal at fertilization is necessary and sufficient for egg activation, which encompasses the completion of meiosis and transition to the embryonic mitotic divisions (Machaca, 2007; Nader et al., 2013). This fertilization induced Ca^2+^ transient has specialized spatial and temporal dynamics that are sufficient to encode the sequential events that need to occur to activate the egg and initiate embryogenesis.

Treatment of porcine oocyte with the GT1b or GD1a gangliosides increased the rate of fertilization and improved oocyte maturation, blastocyst quality and developmental competence of the embryos (Kim et al., 2016; Kim et al., 2020).

### 3.3 Sterols in Oocyte Maturation

Cholesterol rich membrane microdomains are known to be involved in hormonal signal transduction and have been implicated in the control of oocyte maturation (Buschiazzo et al., 2011). In amphibian oocytes, there is conflicting data regarding the role of cholesterol at the PM in regulating oocyte maturation. Cholesterol depletion using methyl-β-cyclodextrin (MβCD) in *Rhinella arenarum* oocytes inhibited oocyte maturation by slowing down the activation of the MAPK pathway (Buschiazzo et al., 2011). Cholesterol depletion was as expected associated with redistribution of raft markers such as Src kinase, caveolin1, and GM1 to non-raft membrane fractions (Buschiazzo et al., 2008; Buschiazzo et al., 2011; Kim et al., 2019). In contrast, others have shown that cholesterol depletion stimulates oocyte maturation by releasing G*α*_s_ and activating the MAPK pathway in *Xenopus laevis* (Sadler and Jacobs, 2004; Sadler et al., 2008). These conflicting data could be due to species differences and/or the fact that studies where cholesterol depletion activated maturation were performed on oocytes from frogs primed by injection of gonadotropins to stimulate oocyte growth. Hence, the cholesterol-dependent modulation of maturation could be a secondary signal that is effective only in pre-stimulated oocytes. Regardless though, an important conclusion from these studies is that modulation of the distribution of lipid raft microdomains impacts oocyte maturation arguing for a signaling role for lipid rafts enriched components in the process. It is important to note that lipid rafts play central roles during *Xenopus* fertilization as well, and this area have been comprehensively reviewed by others (Sato et al., 2006; Stith, 2015).

## 4 Roles of Lipids in Sperm

In contrast to the oocyte, the role of PM lipids during sperm maturation have received significant attention in the literature. This has led to the appreciation of the active remodeling of the sperm lipid composition throughout its maturation journey with important functional consequences. The spermatozoon is a special delivery vehicle of the male DNA to the egg with the capacity to trigger egg activation and embryonic development. The sperm evolved to specifically cater to these functions resulting in a gamete that lacks most typical intracellular organelles such as the Golgi apparatus and ER, and rather possessing a specialized golgi-derive large exocytic component, the acrosome.

The development of the male gamete toward a fertilization competent motile spermatozoon encompasses three broad developmental phases. The first phase is spermatogenesis, which occurs in the male testis and leads to the production of sperm in the seminiferous tubules. The second step is epididymal maturation, during which the sperm within the epididymis acquires motility to be able to swim and fertilize the egg. Interestingly this phase is associated with a significant decrease in sperm phospholipids in the ram, arguing that phospholipids may act as metabolic substrates to provide energy to the sperm (Scott et al., 1967). During the epididymal maturation phase PM lipid and protein composition is also altered in multiple mammalian species (Lenzi et al., 1996). The sperm acquires proteins and small RNAs as well as lipids through extracellular lipid vesicles that are present in the epididymis and vary in their composition in different epididymal regions (Sullivan and Saez, 2013; Shan et al., 2021).

The last step of sperm maturation is capacitation, which confers on the spermatozoa the ability to penetrate and fertilize the egg (Killian, 2004; Kim et al., 2019). This final phase is associated with increased plasma membrane fluidity and the acrosomal reaction, which encompass fusion of the acrosome with the PM thus leading to dramatic changes in the properties and composition of the PM (Lenzi et al., 1996; Gadella et al., 2008). The lipid composition of the sperm PM is important for sperm motility, viability, and fusion with the egg during fertilization (Lenzi et al., 1996). The sperm PM is defined by distinct structural and functional domains such as the cap region above the acrosome that responds specifically to capacitation stimuli. The head is the only region that directly interacts with the egg plasma membrane during fertilization (Lenzi et al., 1996). The lipid composition of the sperm is polarized with distinct lipid content in the sperm head as compared to its tail (Oresti et al., 2011). Finally, the lipid composition of the sperm is also remodeled during its migration in the female reproductive track toward the egg. In the following sections, the roles of different classes of plasma membrane lipids in sperm function will be discussed.

### 4.1 Glycerophospholipids

GPLs are the major constituents of the spermatozoa PM, with PC and PE having the highest percentages. GPLs are also asymmetrically distributed between the inner and outer leaflets of the PM, with PE and PS localizing primarily to the inner leaflet, whereas PC along with sphingomyelin, being enriched in the outer leaflet (Mueller et al., 1994; Lenzi et al., 1996). This asymmetrical distribution may support sperm-egg membrane fusion during fertilization. Membrane fusion requires PM bilayer destabilization and, given their molecular structures and head groups in the inner leaflet, PE and PS favor formation of non-bilayer structures and are therefore more fusion competent compared to PC in the outer leaflet that contains a bulky hydrated head group which is less supportive of membrane fusion (Herrmann et al., 1991; Mueller et al., 1994) (Figure 3B).

The spermatozoa PM also consists of high levels of polyunsaturated fatty acids (PUFA), that derive from GPLs, such as docosahexaenoic acid (DHA), which levels increase during spermatozoa maturation (Lenzi et al., 2000; Aksoy et al., 2006). Many studies have shown that lower levels of DHA and other PUFAs correlate with decreased spermatozoa motility, morphology, concentration, and quality and are thus associated with subfertility and infertility in humans (Zalata et al., 1998; Aksoy et al., 2006; Tavilani et al., 2006; Martínez-Soto et al., 2013). However, it has also been shown that high levels of PUFA in spermatozoa PM can increase susceptibility to ROS, leading to lipid peroxidation, and can decrease membrane fluidity, increase DNA damage, and negatively affect spermatozoa motility (Koppers et al., 2010; Henkel, 2011; Lewis et al., 2013; Aitken et al., 2016). These findings argue for an optimal level of fatty acid content to support sperm physiological functions with extreme levels being associated with negative effects for different reasons.

GPLs have been implicated in activating sperm hypermotility in response to progesterone. The *α*/*β* hydrolase domain-containing protein 2 (ABHD2) was identified as a membrane progesterone receptor in human sperm (Miller et al., 2016; Mannowetz et al., 2017). ABHD2 hydrolyzes 2-arachidonoylglycerol (2-AG) into arachidonic acid and glycerol (Miller et al., 2016). 2-AG inhibits the sperm calcium channel (CatSper). Therefore ABHD2 activation gates open CatSper allowing Ca^2+^ influx into the sperm (Miller et al., 2016) (Figure 3B). The rise in intracellular Ca^2+^ hyperactivates sperm motility to support sperm penetration of the zona pellucida and fertilization (Osman et al., 1989; Blackmore et al., 1990; Baldi et al., 1991).

The abundance of GPLs at the sperm PM have also been linked to releasing sperm from the sperm reservoir within the oviduct. The sperm reservoir stores sperm after insemination through sperm binding to oviduct epithelial cells (Lamy et al., 2017; Ramal-Sanchez et al., 2020). Using oviductal epithelial cell cultures *in vitro*, Ramal-Sanchez et al. showed that progesterone decreases the binding of sperm to these epithelial cells and that the sperm released have decreased levels of Binder of Sperm Proteins (BSP) associated with their PM (Ramal-Sanchez et al., 2020). BSPs are the major protein class in seminal fluid and have been shown to bind to PC and sphingomyelin (Desnoyers and Manjunath, 1992). The decrease in BSPs on the sperm PM allows for their detachment from the oviductal epithelial cells. This detachment was also coupled to an increase in PM fluidity and an increase in both PC and sphingomyelin (Ramal-Sanchez et al., 2020). Therefore, GPLs modulate sperm residency within the oviduct following insemination.

Phospholipase A2 (PLA2) represents a group of enzymes that hydrolyzes GPLs into fatty acids (such as AA or DHA) and lysophospholipids (LPAs, such as LPC) (Yamamoto et al., 2017). PLA2s play important roles in sperm maturation and activation, specifically the secreted PLA2s: sPLA2-III and sPLA2-X (Escoffier et al., 2010; Sato et al., 2010). sPLA2-III enriches the sperm PM with DPA- (docosapentaenoic acid) and DHA-containing PC species, which is thought to promote sperm maturation in the mouse epididymis (Sato et al., 2010). Whereas sPLA2-X is abundantly expressed in the acrosome and participates in sperm activation and fertilization by selectively hydrolyzing DPA/DHA-containing PC species in the sperm PM to release DPA, DHA, and LPC (Escoffier et al., 2010; Murase et al., 2016). These fatty acid products are important for fertilization as shown by the ability of DPA to restore the fertilization ability of sperm from the PLA2-X knockout mice, which are otherwise sub-fertile (Escoffier et al., 2010; Murase et al., 2016; Yamamoto et al., 2017). Therefore, PLA2 products are important for sperm maturation and their fertilization competence (Figure 3B). These findings in conjunction with those from ABHD2 studies highlight the importance of lipase activity in the sperm with PM GLPs as substrates to produce lipid products that are critical for sperm activation.

Therefore, GPLs are involved in many aspects of sperm physiology ranging, including sperm quality, fertilization competency, residency in the sperm reservoir, and hypermotility. This broad spectrum of sperm related functions highlights the importance of GPL biology in sperm physiology.

### 4.2 Sphingolipids

Gangliosides have been shown to be involved in sperm motility, specifically protecting the sperm PM from reactive oxygen species (ROS) damage. When added exogenously, GT1b and GD1b are adsorbed to the sperm surface and reduce the levels of superoxide anions in human sperm (Tyurin et al., 1992; Kim et al., 2019). In addition, ganglioside micelles have been shown to attach to the ejaculated sperm PM and protect it from ROS-induced damage (Hwang et al., 2015; Kim et al., 2019). GM3 is suggested to play a role in sperm maturation as it is detected in both spermatocytes and spermatids (later stages of spermatozoa development) but not in spermatogonia (first stage of spermatozoa development) in adult rat seminiferous tubules (Jung et al., 2001).

During capacitation of rat, mouse, and bovine sperm, GM1 shifts its localization from cholesterol-poor regions on the PM of the sperm head to cholesterol-rich lipid rafts overlying the acrosome (Roberts et al., 2003; Selvaraj et al., 2007). In ejaculated boar spermatozoa, cholesterol depletion by MβCD led to the redistribution of GM1 from the sperm tail to the sperm head (Dunning et al., 2015; Kim et al., 2019). Moreover, by assessing changes in GM1 distribution in response to progesterone-induced acrosomal exocytosis (AE), patterns reflecting the response of murine sperm populations to capacitating stimuli and GM1 localization were established. These data suggest that GM1 localization can be used as a diagnostic tool to assess the response of sperm to capacitation stimuli and/or acrosome exocytosis (Selvaraj et al., 2007). These findings have been translated to clinical application with the development of a test called Cap-Score™ Sperm Function Test (Cap-Score), which was designed to assess sperm capacitation based on GM1 localization patterns of (Moody et al., 2017).

### 4.3 Cholesterol and Lipid Rafts

As a main component of the sperm PM, cholesterol plays an important role in sperm motility, maturation, and capacitation (Liu and Ding, 2017). During sperm maturation, cholesterol content significantly decreased by approximately 50% in several mammalian species such as rat, mouse, and hamster (Parks and Hammerstedt, 1985; Hall et al., 1991; Awano et al., 1993; Rejraji et al., 2006; Saez et al., 2011). This drop in cholesterol content causes a reduction in the cholesterol:phospholipid ratio that leads to an increase in membrane fluidity, which facilitates capacitation and sperm-egg membrane fusion during fertilization (Whitfield et al., 2015) (Figure 3B).

During sperm capacitation, the modulation of cholesterol efflux by proteins such as NPC2 (Niemann-Pick type C2) was also shown to increase PM permeability to Ca^2+^ and bicarbonate ions (Lee and Storey, 1986; Visconti et al., 1995; Cross, 1998). The influx of these ions leads to the activation of soluble adenylyl cyclase, increase in cAMP, activation of protein kinase A (PKA), and phosphorylation of Src kinase (Visconti et al., 1995; Xie et al., 2006). In addition, cholesterol is a critical component of PM lipid rafts, which serve as signaling hubs in sperm (Travis et al., 2001). During bull spermatozoa maturation, the GPI-anchored protein P25b, which is needed for zona pellucida recognition by the spermatozoa, was found to be transferred to lipid rafts via small lipid vesicles from epididymal epithelium (Girouard et al., 2009). Furthermore, it has been shown in animal models and clinical studies that a rise in sperm cholesterol due to high body mass index and obesity led to a decrease in spermatozoa motility, premature acrosome reaction, and overall reduced semen quality (Schisterman et al., 2014; Andersen et al., 2015; Liu and Ding, 2017).

The reduction of cholesterol during capacitation is associated with its lateral redistributions with implications on enrichment of lipid raft domains (Van Gestel et al., 2005). Lipid raft proteins, caveolin and flotillin, redistribute to the anterior head of the sperm during capacitation (Van Gestel et al., 2005). Interestingly, these changes correlate with redistribution of SNARE proteins that mediate the acrosome reaction. Two target SNAREs (t-SNARE) syntaxin 1 and 2, have been found to localize primarily to the plasma membrane in mammalian sperm, whereas the vesicle SNARE (v-SNARE) VAMP is enriched in the outer acrosomal membrane (Tsai et al., 2007). Capacitation is associated with redistribution of SNARE proteins to the sperm head apical ridge, which is the site of zona binding that precedes acrosome exocytosis. This argues that cholesterol efflux and raft reorganization prepare the sperm for the acrosome reaction (Tsai et al., 2010). Electron microscopy coupled to proteomics analyses further argues that the acrosome reaction is associated with exposure on the sperm head of acrosomal proteins that are likely to bind glycoproteins on the zona thus supporting sperm-egg interactions (Tsai et al., 2012).

Changes in sperm lipids have been implicated in fertilization by multiple studies. This area has been carefully reviewed recently by others (Stith, 2015; Shan et al., 2021), so we will not reiterate those summaries here.

### 4.4 Fatty Acids

Lipidomics approaches allow comprehensive analyses of global lipid profile at the cellular level and have been used to define the lipid composition of sperm in various species (Evans et al., 2021). For example, lipidomic studies in the horse identified for the first time the presence of (O-acyl)-omega-hydroxy-fatty acids (OAHFA) in the sperm head but not the tail. OAHFA can be quite long (up to 52 carbons) and has amphiphilic and surfactant properties, which may play roles in sperm docking and prefusion (Wood et al., 2016). In dogs lipidomics analyses were performed on sperm collected from different segments of the cauda epididymis. The concentrations of saturated, monounsaturated, and polyunsaturated fatty acids were higher in sperm isolated from the cauda epididymis as compared to caput and corpus sperm (Ramos Angrimani et al., 2017). Cauda sperm are more mature and acquire these changes in lipid profiles in conjunction with increased membrane integrity and sperm motility in preparation for fertilization.

### 4.5 Sulfogalactosylglycerolipid (SGG, Seminolipid)

In vertebrate membranes, glycolipids are typically synthesized through the addition of a glycosidic moiety to ceramide, with the exception of SGG (Tanphaichitr et al., 2018). SGG is synthesized exclusively in mammalian testicular germ cells and represents a significant proportion of total lipids with the vast majority of SGG having the structure 1-*O*-hexadecyl, 2-*O*-hexadecanoyl, 3-3′-sulfogalactosyl glycerol (Flesch and Gadella, 2000; Tanphaichitr et al., 2018). It is required for spermatogenesis, the acrosome reaction, and gamete fusion during fertilization. Interestingly, SGG is concentrated in the apical ridge region of the sperm head in its sulfated form in ejaculated sperm, and as is the case for other glycolipids, it localizes to the outer leaflet of the PM (Gadella et al., 1993; Vos et al., 1994). Apical SGG prevents the acrosome reaction by stabilizing the bilayer and by reducing Ca^2+^ influx (Flesch and Gadella, 2000). Following capacitation SGG moves to the equatorial region of the sperm head, which is the region that mediates lateral binding to the oolemma (Flesch and Gadella, 2000). SGG interacts with seminolipid immobilizing protein (SLIP), which likely modulates SGG dynamics through interactions with the glycocalyx (Flesch and Gadella, 2000). SLIP in turn plays a role in zona pellucida interactions (Tanphaichitr et al., 1998). As an anionic glycolipid the free SGG may bind to the zona through electrostatic interactions as well (Flesch and Gadella, 2000). The biosynthetic pathway mediating SGG synthesis and metabolism has been elucidated and male knockout mice in genes required for SGG biosynthesis are infertile due to arrested spermatogenesis at the primary spermatocyte stage (Tanphaichitr et al., 2018). SGG is an ordered lipid that is enriched in lipid rafts and this property helps mediate its differential localization during sperm maturation on the PM (Tanphaichitr et al., 2018). As is the case with glycolipids in general, SGG is involved in cell adhesion through its binding to multiple cell surface and extracellular proteins (Tanphaichitr et al., 2018).

## 5 Conclusion and Future Directions

In conclusion, lipids are involved at multiple levels of gamete maturation. Not only do lipids participate in the structural, organizational, and biophysical properties of the cell membrane but they have also been shown increasingly to play roles in lipid-protein interactions and signal transduction pathways. The majority of the studies to date have focused on changes in the amount and composition of gamete lipids. Clearly though there is significant and intricate crosstalk between different lipid signaling pathways. Thus, more global changes in lipid composition through a detailed time course using lipidomics approaches should help the field in better integrating the various changes in PM lipids toward defining their roles in mediating gamete function. In addition to increasing the depth of the analysis and its time resolution, a daunting challenge in the future will be defining the physiological signaling pathways associated with dynamic lipid changes. The combination of lipidomics with model systems should help in increasing the throughput on that front.
